# Pulmonary mixed squamous cell and glandular papilloma in the elderly: a case report and literature review

**DOI:** 10.3389/fonc.2025.1606426

**Published:** 2025-12-16

**Authors:** Panpan Zhang, Yuan Wang, Chao Huang, Yanyan Ji, Xiaoqing Han

**Affiliations:** 1 Department of Respiratory Medicine, North China University of Science and Technology Affiliated Hospital, Tangshan, China; 2 Department of Respiratory Medicine, Tangshan Gongren Hospital, Tangshan, China

**Keywords:** papilloma, squamous cell, lung neoplasms, aged, bronchi, case reports

## Abstract

Mixed squamous cell and glandular papilloma (MSCGP) is a rare benign lung tumor, which mostly occurs in the central airway and primarily affects middle-aged and elderly individuals. Due to the limited understanding of the clinical characteristics of elderly patients, clinicians often lack a comprehensive grasp of MSCGP, leading to misdiagnosis. Therefore, in this article, we report a case of an elderly patient with central MSCGP and provide a comprehensive review of the relevant literature on elderly patients with MSCGP, aiming to improve the diagnostic rate of this disease.

## 1 Introduction

Mixed squamous cell and glandular papilloma (MSCGP) of the lung is an extremely rare benign tumor that mainly affects the elderly. Currently, there is a lack of review studies on the clinical characteristics of MSCGP in the elderly population. Clinicians have an insufficient understanding of the clinical features of this tumor, which often leads to misdiagnosis as mucoepidermoid carcinoma, adenocarcinoma, or other diseases. This has serious consequences for public health. This study reports a case of an elderly male patient with MSCGP of the lung and provides a literature review focused on the elderly population.

## 2 Case report

A 62-year-old man presented with an unexplained cough and was ultimately diagnosed with MSCGP of the lung based on chest computed tomography and bronchoscopy. One month earlier, the patient developed an irritating dry cough with no other relevant symptoms. To further clarify the cause of the cough, the patient was admitted to the respiratory department of a local hospital. Chest radiography revealed no abnormalities. Lung function tests suggested reductions in the volume of 1 s and maximum mid-expiratory flow rate, and the bronchodilation test was negative; thus, the cough was attributed to bronchitis. Although the patient was given antibacterial and antitussive treatment for bronchitis, the symptom of irritating dry cough did not improve significantly. Subsequently, he was admitted to the respiratory department of North China University of Science and Technology Affiliated Hospital for further evaluation.

**Figure 1:**
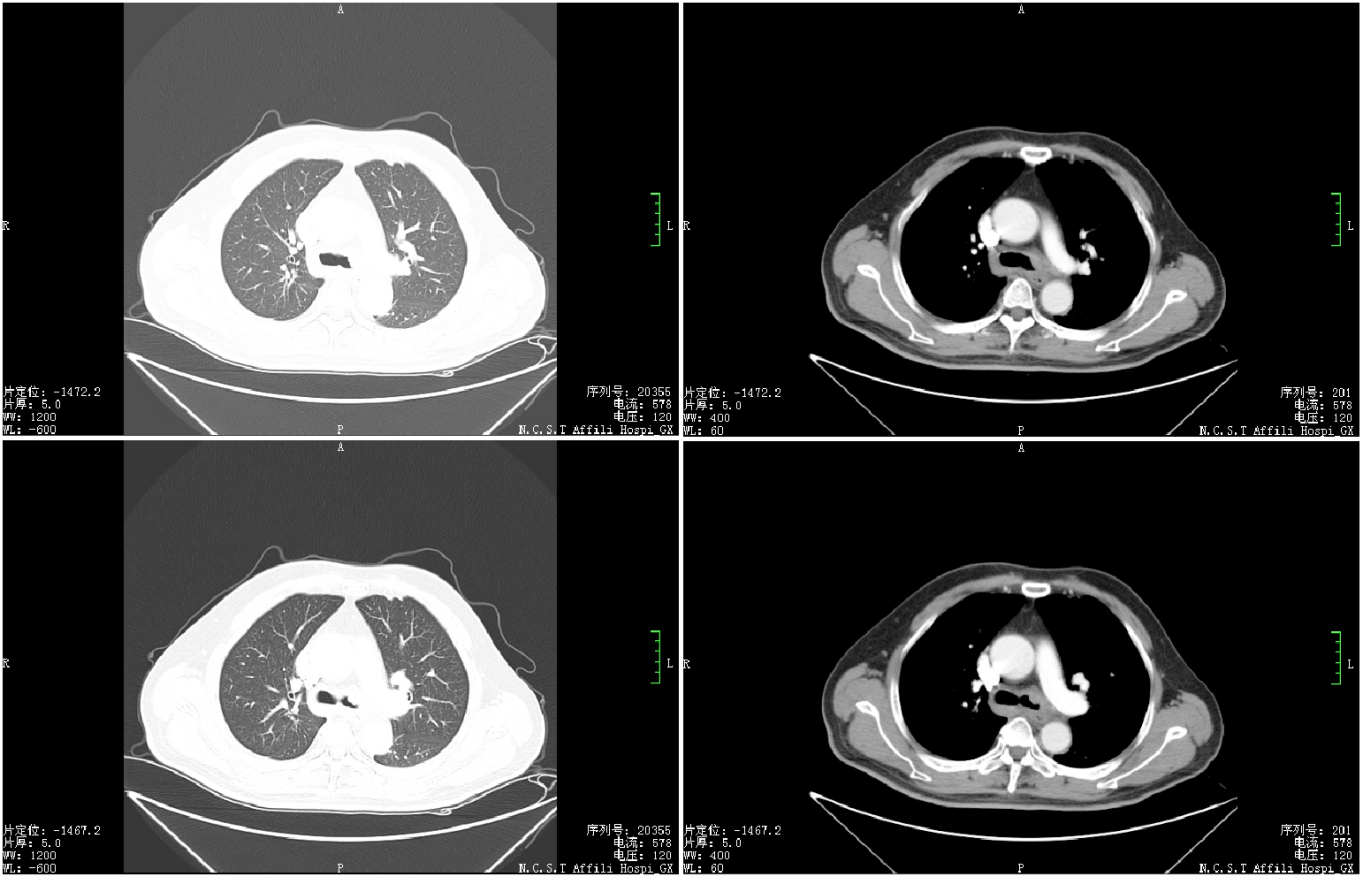
Chest computed tomography scan showing a left main bronchial mass.

There was no other past history of disease except for type 2 diabetes. However, the patient had 50 years of smoking history. Physical examination revealed lower respiratory sounds in the left lung, and no obvious abnormalities were found in the rest of the the physical examination. Chest computed tomography performed at our hospital indicated the growth of a mass in the left main bronchus (**Figure 1**).

**Figure 2:**
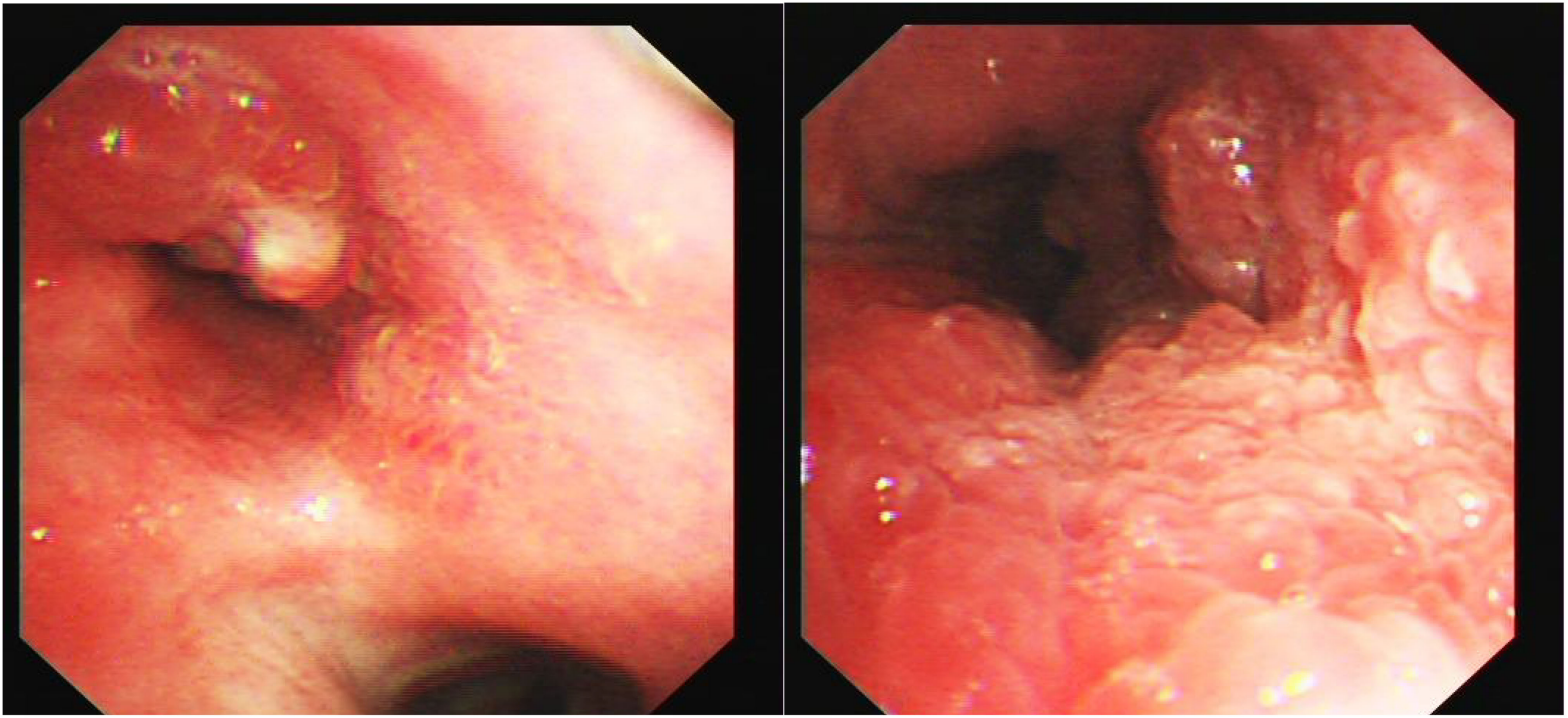
Bronchoscopy showing a cauliflower-like neoplasm invading the carina.

**Figure 3:**
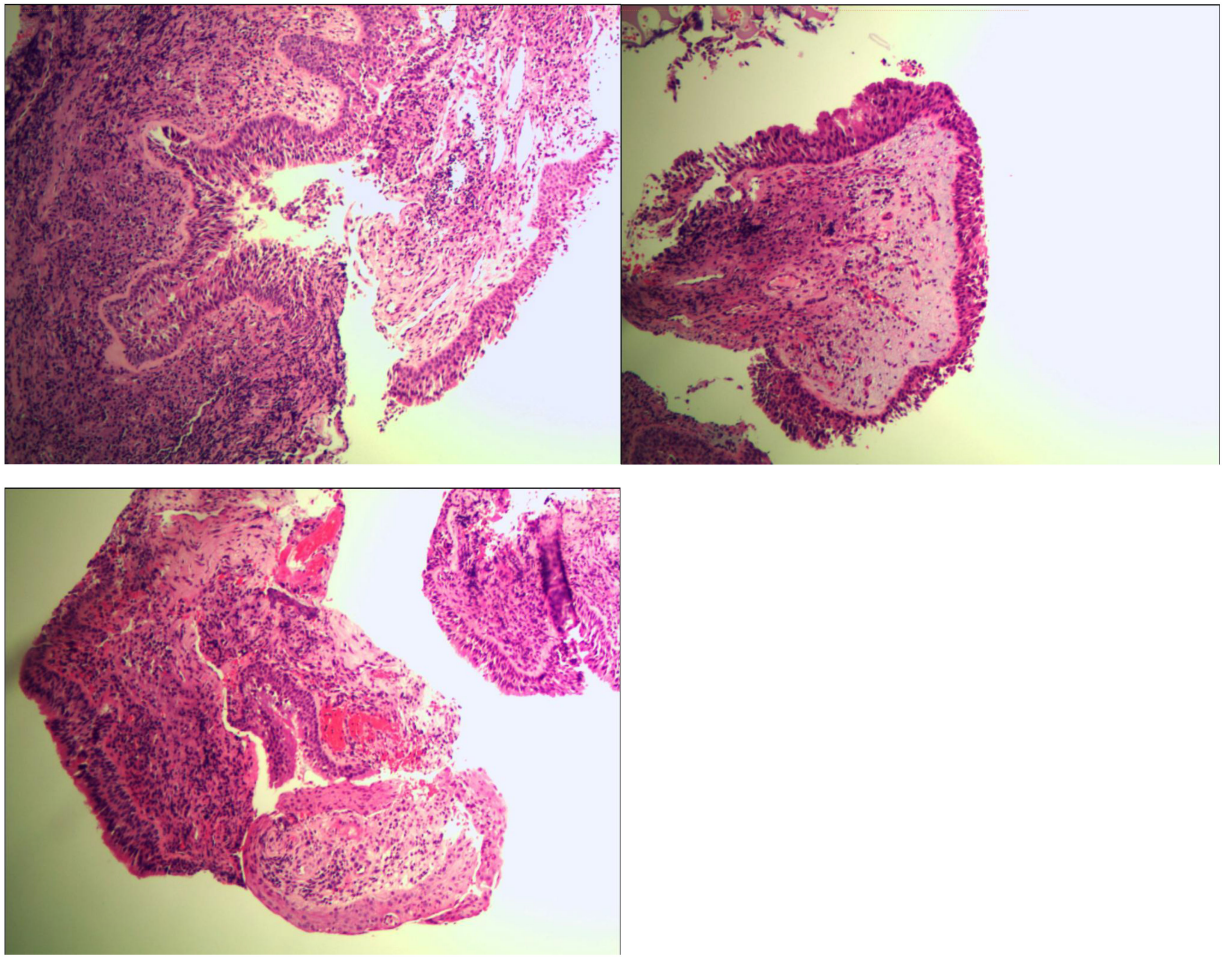
Pathological result of bronchoscope forceps showing mixed squamous cell and glandular papilloma of the lung. Hematoxylin and eosin staining, original magnification: ×100.

Bronchoscopy revealed a cauliflower-like neoplasm invading the carina of the left main bronchus (**Figure 2**). Consequently, we performed bronchoscopy biopsy, and the pathological results of bronchoscopy forceps were analyzed. Histological findings revealed papillary structures in the tumor area against a mucinous background. The center of the papillae had a fibrovascular core, in which a large number of lymphocytes and eosinophils were found. The surface was covered with squamous and glandular epithelium, including pseudostratified ciliated or non-ciliated columnar epithelium and mucinous columnar epithelium, with no cellular dysplasia or necrosis observed (**Figure 3**).

Immunohistochemistry results indicated that squamous epithelial cells were positive for cytokeratin 5/6 (CK5/6) and p40 expression, whereas glandular epithelial cells were positive for CK7 and thyroid transcription factor 1 (TTF-1) expression. The Ki-67 proliferation index of the tumor was 3%. Laboratory findings revealed normal serum carcinoembryonic antigen (CEA) levels, blood routine test results, C-reactive protein concentration, and erythrocyte sedimentation rate. Therefore, the patient was diagnosed with MSCGP of the lung. Because the MSCGP of the lung invaded the carina, radical resection was not performed. The patient underwent bronchoscopic interventional treatment, which included argon high-frequency electroknife resection and CO_2_ cryoablation. The patient refused further treatment. No evidence of recurrence was observed after 6 months of follow-up.

## 3 Discussion

The lung MSCGP is an extremely rare benign tumor composed of bidirectionally differentiated squamous and glandular epithelial cells (1). It was first identified by Flieder in 1998 (2). The distribution of MSCGP is from the third to the sixth decade of life (3).

In 2015, the World Health Organization classified solitary endobronchial papillomas into three categories according to different epithelial components: squamous epithelial papilloma, glandular papilloma, and MSCGP; among them, MSCGP is the rarest type (4). The clinical manifestations of pulmonary MSCGP are diverse and not specific, and the corresponding symptoms (e.g.,irritating dry cough, sputum, hemoptysis, and chest pain) are often caused by mass obstruction and oppression (5).

A comprehensive search of PubMed, CNKI, and Wanfang databases case reports and case series published between 2010 and 2024.The language limit was English and Chinese.The search used keywords including “MSCGP” or “Solitary endobronchial papilloma.” Our search focused exclusively on patients aged ≥60 years who met the diagnostic criteria for MSCGP.

The characteristics of MSCGP described in previous reports are shown in **Table 1** (6–33). Most elderly patients with MSCGP included in the studies were from Asia and Europe. This indicates that clinicians in these countries attach importance to the diagnosis of MSCGP. It has been previously suggested that tumors are more commonly found in men (male-to-female ratio: 16:5), and most patients have a history of smoking.

**Table 1:** . Summary of published cases of mixed squamous cell and glandular papilloma of the lungs in elderly patients.

Case no.	Reference	Country	Age (years)/ sex	Clinical symptoms	Max. diameter (cm)	Classification of lesion location	Treatment	Smoking history	Blood tumor markers	Histological findings			Immunohistochemistry	Follow-up
										Mucous component	Ciliated columnar cells	Papillary structure		
1	Lu et al. (6)	China	63/F	Cough, hemoptysis	2.0	Central type	Left upper lobe resection	NM	NM	+	+	+	p63 (+), Ki-67: 5%	6 months, A&W
2	Lu et al. (6)	China	73/F	Cough, bloody phlegm	3.1	Central type	Left upper lobe resection	NM	NM	+	+	+	p63 (+), Ki-67: 5%	5 months, A&W
3	Toshiharu et al. (7)	Japan	64/M	Asymptomatic	6.0	Peripheral type	Left lower lobe resection	43 PY	Elevated CEA and SCC levels	+	+	+	NM	NM
4	Dong et al. (8)	China	64/F	Chest pain	1.3	Peripheral type	Right lower lobe wedge resection	No	NM	+	−	+	CK5/6, CK7, CK19, CAM5.2, CK34βE12, and TTF-1 (+)	24 months, A&W
5	Yoshiki et al. (9)	Japan	60/M	Asymptomatic	1.8	Peripheral type	Right middle lobe resection; right middle lobe partial resection	40 PY	Elevated CEA levels; normal NSE levels	+	+	+	CK5/6, CK7 (+), TTF-1, CK20, p40, and CEA (−), Ki-67 (+)	3 months, A&W
6	Ju et al. (10)	Japan	64/F	Asymptomatic	4.0	Peripheral type	Left lower lobe wedge resection	NM	NM	+	−	+	CK5/6, CK7, and p63 (+), Ki-67: 1%	18 months, A&W
7	Chen et al. (11)	China	68/F	Chest distress, wheezing	3.0	Peripheral type	Right lower lobe resection	NM	NM	+	+	+	CK5/6, CK7, TTF-1, CKH, CHL, and p63 (+)	6 months, A&W
8	Feng et al. (12)	China	63/F	Cough, expectoration	2.5	Peripheral type	Right upper lobe wedge resection	NM	NM	+	+	+	CK7, TTF-1, and p63 (+)	20 months, A&W
9	Jiro et al. (13)	Japan	68/F	Asymptomatic	1.0	Peripheral type	Left upper lobe wedge resection	22 PY	NM	+	+	+	CK5/6 and p40 (+)	6 months, A&W
10	Ryo et al. (14)	Japan	72/M	Asymptomatic	2.0	Central type	Right upper lobe resection	60 PY	Normal CEA, NSE, and SCC levels	+	+	+	CK5, p40, p53, and Ki-67 (+)	12 months, A&W
11	Kei et al. (15)	Japan	76/M	Asymptomatic	1.1	Peripheral type	Right lower lobe resection	40 PY	Elevated CEA and SCC levels	+	+	+	CK5/6, CK7, and p63 (+), CK20 (−), Ki-67: 5%	30 months, A&W
12	Liu et al. (16)	China	70/M	Cough, expectoration	4.0	Peripheral type	Left lower lobe resection	40 PY	NM	+	+	+	CK5/6 and CK7 (+), CEA, TTF-1, CK20, villin, and CDX-2 (−), Ki-67: 1–2%	13 months, A&W
13	Feng et al. (17)	China	60/M	Cough, expectoration, hemoptysis	1.1	Peripheral type	Right lower lobe wedge resection	NM	Normal CEA, NSE, and SCC levels	+	+	+	CK5/6, CK7, TTF1, p63, p40, pCK, and Napsin-A (+), CK20 and p16 (−), Ki-67: 3%	6 months, A&W
14	Feng et al. (17)	China	65/F	Chest distress	0.8	Peripheral type	Right lower lobe wedge resection	20 PY	Normal CEA, NSE, and SCC levels	+	+	+	CK5/6, CK7, TTF1, p63, p40, pCK, and Napsin-A (+), CK20 and p16 (−), Ki-67: 3%	10 months, A&W
15	Li et al. (18)	China	67/F	Chest pain	2.0	Peripheral type	Right upper lobe wedge resection	NM	NM	+	+	+	CK7, TTF-1, villin, CK20, and Napsin-A (+), Ki-67: 3%	35 months, A&W
16	Dong et al. (19)	China	64/F	Cough, chest pain	5.0	NM	Right lower lobe resection	NM	NM	+	+	+	CK5/6, CK7, TTF-1, p63/p40, and BRAF (+), CK20 and Napsin-A (−), Ki-67: 2%	61 months, A&W
17	Dong et al. (19)	China	70/M	Cough, bloody phlegm	3.1	NM	Left lower lobe resection	NM	NM	+	+	+	CK5/6, CK7, TTF-1, p63/p40, and BRAF (+), CK20 and Napsin-A (−), Ki-67: 2%	6 months, A&W
18	Chen et al. (20)	China	64/M	Asymptomatic	2.3	Central type	Surgical resection	NM	NM	+	+	+	CK7, TTF-1, p63, p40, and CEA (+), Ki-67: <1%	6 months, A&W
19	Chen et al. (20)	China	64/F	Asymptomatic	1.0	Peripheral type	Surgical resection	NM	NM	+	+	+	CK7, TTF-1, p63, p40, and CEA (+), Ki-67: <1%	12 months, A&W
20	Chen et al. (21)	China	63/F	Cough	3.0	Peripheral type	Dorsal segment of right lower lobe resection	NM	Normal CEA and NSE levels	+	+	+	CK5/6, CK7, TTF-1, CK19, CAM5.2, CK34βE12, and p40 (+), Ki-67: 5%	5 months, A&W
21	Wang et al. (22)	China	65/M	Cough, expectoration	NM	NM	NM	NM	NM	−	−	+	TTF-1, Napsin-A, MUC5AC, p40 (+), Ki-67: 2~5%	NM
22	Wang et al. (22)	China	70/M	Cough, expectoration	NM	NM	NM	NM	NM	−	−	+	TTF-1, Napsin-A, MUC5AC, p40 (+), Ki-67: 2~5%	NM
23	Xiao et al. (23)	China	74/M	Asymptomatic	0.7	NM	Surgical resection	30 PY	NM	+	+	+	CK5/6 and p63 (+), p16 and TTF-1 (−), Ki-67: 3%	16 months, A&W
24	Konaka et al. (24)	Japan	76/F	Asymptomatic	2.1	NM	Right lower lobe resection	NM	Normal CEA and NSE levels	−	−	+	NM	48 months, A&W
25	Iteeka et al. (25)	United Kingdom	77/F	Weight loss	3.5	Peripheral type	Right lower lobe wedge resection	NM	NM	+	−	+	p63 and TTF-1 (+)	NM
26	Che et al. (26)	China	74/F	Asymptomatic	3.0	Central type	Left lower lobe resection	NM	NM	+	+	+	CK7, TTF-1, MUC5AC, CK5/6, p63, and p40 (+), Napsin-A, CK20, CDX-2, p16, ALK-D5F3, and BRAFV600E (−), Ki-67: 5%	9 months, A&W
27	Che et al. (26)	China	66/M	Chest distress, chest pain	1.7	Central type	Right upper lobe resection	NM	NM	+	+	+	CK7, TTF-1, MUC5AC, CK5/6, p63, p40 (+), Napsin-A, CK20, CDX-2, p16, ALK-D5F3, and BRAFV600E (−), Ki-67: 5%	24 months, A&W
28	Haga et al. (27)	Japan	76/F	Asymptomatic	2.5	Peripheral type	Right lower lobe resection	NM	Elevated CEA and SCC levels	−	−	+	NM	6 months, A&W
29	Sato et al. (28)	Japan	85/M	Asymptomatic	1.2	NM	Right lower lobe wedge resection	NM	Normal CEA and SCC levels	+	+	+	CK5/6 and p40 (+), TTF-1, and Napsin-A (−)	12 months, A&W
30	Wang et al. (29)	China	67/M	Asymptomatic	1.7	Peripheral type	Left lower lobe wedge resection	NM	Normal CEA and NSE levels	+	+	+	p40, p63, CK5/6, and TTF-1 (+), Napsin-A (−), Ki-67: <2%	12 months, A&W
31	Cao et al. (30)	China	67/M	Left limb weakness	1.5	Central type	Transbronchial resection of bronchoscopic masses	50 PY	NM	−	−	+	CK5/6, CK7, TTF-1 (+), p63, and p40 (+), Napsin-A (−), Ki-67: 1%	2 months, A&W
32	Nitanda et al. (31)	Japan	82/M	Bloody phlegm	4.3	Peripheral type	Left lower lobe resection	60 PY	Elevated CEA levels	+	+	+	NM	26 months, A&W
33	Zhang et al. (32)	China	69/F	Asymptomatic	2.6	Central type	Right lower lobe resection	NM	Elevated CEA levels	−	−	−	NM	NM
34	Huo et al. (33)	China	61/M	Cough, expectoration	Diffuse	Central type	Transbronchial resection of bronchoscopic masses	30 PY	Normal CEA, SCC, and NSE levels	−	−	−	CK5/6, CK7, TTF-1, p63, p40, and CD34 (+), Ki-67: 5%	14 months, A&W

A&W, alive and well; ALK, anaplastic lymphoma kinase; CEA, carcinoembryonic antigen; CK, cytokeratin; F, female; M, male; Max., maximum; MUC5AC, mucin 5AC; NM, not mentioned; NSE, neuron-specific enolase; PY, pack-years; SCC, squamous cell carcinoma antigen; TTF-1, thyroid transcription factor. + and (+), positive; - and (-),negative.

However, our literature review summarizing the clinical features of MSCGP in elderly patients in the past 14 years revealed a male-to-female ratio of 1:1 (i.e., 17 males and 17 females), indicating that there is no sex difference in the incidence of the disease. It also confirmed that smoking history is related to MSCGP in the elderly, and the level of the smoking index may affect the incidence of MSCGP in this population. Moreover, we found that MSCGP in the elderly not only occurs more frequently in the central airway, but also more often in the peripheral airway. However, previous studies have shown that MSCGP in elderly individuals is more prevalent in the central airways,the reason for this inconsistency remains unclear.

Among the 34 elderly patients with MSCGP, 19 had a variety of symptoms, among which cough (42%) was the most common,followed by hemoptysis or bloody phlegm (19%), chest pain (15%), chest distress (12%), wheezing (4%), weight loss (4%), and left limb weakness (4%). Cough, hemoptysis, and chest pain are the main symptoms in elderly patients with MSCGP. Previous studies have also demonstrated that cough, hemoptysis, and dyspnea are common symptoms of MSCGP in adults (3); nevertheless, the main symptoms in the elderly were not different from those observed in adult patients. Furthermore, 15 patients diagnosed with MSCGP through physical examination did not develop any symptoms. This shows that the onset of MSCGP is insidious and not characterized by typical clinical manifestations; hence, it is easily overlooked by clinicians.

Furthermore, we found that the maximum diameter of MSCGP lesions mostly ranged from 1 cm to 5 cm in elderly patients. Among the 34 elderly patients with MSCGP, only one patient showed diffuse growth in the central airway. The patient reported in this study also showed diffuse growth in the central airway. For tumor tissues that diffuse along the central airway, radical resection of the lobe or segment of the lung is not possible. Therefore, only interventional treatments under bronchoscopy such as argon plasma coagulation, cryotherapy, and laser therapy can be performed. According to our literature review, radical resection of the lobe or segment of the lung has been adopted in most cases reported to date. Regarding prognosis after resection, the postoperative follow-up time exceeded 2 months, with the longest reaching 61 months, indicating a longer survival period in elderly patients.

Typically, the histological morphology of MSCGP is mainly composed of a mixture of papillae with vascular cores, which consist of glandular epithelial cells covering the surface and squamous epithelial cells and basal cells beneath them (34). In most of the included elderly MSCGP cases, histological features such as mucous components, ciliated columnar cells, and papillary structures were observed. MSCGP needs to be differentiated from malignant tumors such as mucinous adenocarcinoma and adenosquamous carcinoma. When the background of MSCGP is rich in mucous components, with glandular epithelium as the main component and relatively few squamous epithelial components, it can easily be misdiagnosed as mucinous adenocarcinoma.

Pulmonary mucinous adenocarcinoma shows invasive growth, obvious stromal reactions, significant cellular dysplasia, and a high Ki-67 proliferation index. When MSCGP has a similar mixed ratio of glandular epithelium and squamous epithelium, it is highly likely to be misdiagnosed as a pulmonary adenosquamous carcinoma, particularly under low-power microscopy. Under high-power microscopy, cellular and nuclear dysplasia of the squamous epithelium and glandular epithelium was observed, keratin pearls and intercellular bridges in the squamous epithelial areas were identified, and an accurate diagnosis was eventually made based on the immunohistochemistry results. Immunohistochemical detection can improve the diagnostic accuracy of MSCGP. Among the 34 elderly patients with MSCGP, 29 underwent immunohistochemical detection. The results showed that CK5/6, CK7, TTF-1, p63, and p40 were positively expressed in both glandular and squamous epithelial cells of MSCGP. Among these markers, TTF-1 and CK5/6 were expressed in glandular epithelial cells and squamous epithelial cells, respectively. Moreover, Ki-67 levels were detected in most cases; the analysis showed that the maximum proliferation index of tumor cells did not exceed 5%. Therefore, Ki-67 detection helps distinguish between benign and malignant tumors, underscoring the importance of immunohistochemistry in the diagnosis of MSCGP.

It has been reported that 71.4% of patients with MSCGP have elevated levels of blood tumor markers (35). However, some reports indicated that the serum levels of CEA and squamous cell carcinoma antigen (SCC) were normal, and the number of tumor cells secreting these factors was too low to elevate their serum levels (36, 37). Among the 34 elderly patients with MSCGP included in our review, only 14 underwent blood tumor marker testing. Three patients had simultaneous elevation of CEA and SCC antigen levels, three patients had elevated CEA levels alone, and the remaining eight patients had normal levels of blood tumor markers. The results revealed that CEA and SCC antigen levels increased in elderly patients with MSCGP. Therefore, blood tumor marker tests should be routinely performed in such patients. The increase in blood CEA and SCC antigen levels has a certain reference value for diagnosis. In clinical practice, attention should be paid to the combined increase in CEA and SCC antigen levels.

In summary, through our literature review, we conducted a comprehensive and thorough examination of the clinical characteristics of MSCGP in elderly patients. This analysis may enable the medical staff to recognize that MSCGP rarely occurs and can imitate malignant lesions. This information should be considered in the differential diagnosis of elderly patients in the future to improve the diagnostic rate of MSCGP.

## Data Availability

The original contributions presented in the study are included in the article/supplementary material. Further inquiries can be directed to the corresponding author.
